# Modulatory Effects of Pre- and Post-Drought Root Application of Melatonin on the Antioxidant Defense in Young Wheat Plants

**DOI:** 10.3390/ijms27156838

**Published:** 2026-07-30

**Authors:** Elena Shopova, Zornitsa Katerova, Irina Vaseva, Liliana Brankova, Dessislava Todorova, Tsvetina Nikolova, Martin Iliev, Iskren Sergiev

**Affiliations:** Institute of Plant Physiology and Genetics, Bulgarian Academy of Sciences, Acad. Georgi Bonchev Street, Bldg. 21, 1113 Sofia, Bulgaria; kostei@abv.bg (E.S.); vaseva@bio21.bas.bg (I.V.); lbrankova@abv.bg (L.B.); dessita@bio21.bas.bg (D.T.); tnikolova00@bio21.bas.bg (T.N.); martin.vl.iliev@gmail.com (M.I.); iskren@bio21.bas.bg (I.S.)

**Keywords:** drought, melatonin, enzymatic antioxidant activity, transcript profiling, glutathione, *Triticum aestivum* L.

## Abstract

Melatonin is a naturally occurring compound that regulates many aspects of plant growth and development. Recently, its stress-protective potential has been extensively studied. This study investigates the effect of exogenous melatonin on non-enzymatic antioxidants, gene expression, and activity of key antioxidant enzymes in young winter wheat plants subjected to 5 days of drought. Melatonin was root-supplemented 24 h before or after the stress. The parameters were analyzed in the leaves of two Bulgarian cultivars at the end of drought, and after recovery. Drought activated both enzymatic and non-enzymatic antioxidant defense in both cultivars, with distinct responses reflecting their tolerance. The drought-tolerant cv. Gines showed marked increase in total phenolics, thiol containing compounds, catalase (CAT) and glutathione reductase (GR) activities, and catalase (*CATA*, *CAT3*) and class III peroxidase (*POX2*) transcript levels. The less tolerant cv. Fermer exhibited more limited induction, primarily involving *CATA*, *CAT3* and *GR* transcripts and the glutathione pool. Melatonin pre-treatment generally attenuated drought-induced antioxidant responses. This effect was more pronounced in cv. Fermer, where the most drought-responsive parameters were also the most alleviated by melatonin, whereas in cv. Gines only *CATA* expression, and CAT and GR activities were significantly influenced, suggesting cultivar-dependent modulation of antioxidant systems by melatonin. During recovery, both pre- and post-drought melatonin applications produced comparable effects on the antioxidant defense. The post-treatment selectively enhanced the studied transcripts in a cultivar-specific manner.

## 1. Introduction

Climate change has emerged as a worldwide issue. Drought is a major abiotic stress factor affecting various plant physiology parameters that disturbs multiple biochemical and molecular biology processes. It hinders each stage of plant ontogenesis and has a devastating effect on plant organisms under environmental extremes. Research on stress mitigation tools in drought-affected wheat becomes increasingly important due to the ongoing climate changes [1,2,3,4]. Wheat is an important staple crop that usually tolerates mild drought stress but under prolonged water shortage significantly compromises its quality and productivity. Stress factors, including drought, provoke overproduction of reactive oxygen species (ROS), activating the antioxidant defense system that comprises both non-enzymatic and enzymatic antioxidants [5,6]. The main components of the enzymatic antioxidant defense are the families of superoxide dismutase (SOD), glutathione S-transferase (GST), catalase (CAT), peroxidase (POX) and enzymes from the ascorbate–glutathione cycle [5,6]. Non-enzymatic antioxidants include alpha-tocopherol, proline, glycine betaine, carotenoids, ascorbate, phenolic and thiol groups containing compounds, glutathione, etc. [6,7,8,9]. It is well known that plants successfully overcome the impact of diverse abiotic factors when the stress does not surpass their native adaptive mechanisms. However, when the intrinsic plant defense systems are unable to provide adequate protection, a rational environmentally safe approach to improve drought resilience through support of the endogenous antioxidant capacity is needed. It could be achieved by the so-called priming or application of suitable biostimulants.

Priming, or preliminary exposure to mild stress, is one of the common strategies reported to modulate water deficit effects as well as other stress consequences in different plants [1,3,4,10]. In general, priming could be done on seeds, seedlings, aerial or underground plant parts. Priming with plant hormones or growth regulators has shown a good potential for agricultural application [3,11]. Seed priming is the most commonly performed hormone priming method [11].

Biostimulant formulations are another promising strategy to counteract various abiotic stress effects in plants, including water shortage. They are classified in different groups such as humic substances, seaweed extracts, microorganisms, hydrolyzed proteins and amino-acid-comprising products. Some of them contain physiologically active phytohormones, plant growth regulators or various biologically active compounds that are capable to modify stress-protective reactions [3,12,13]. The application of biostimulants might be performed not only before the stress, as in the case of priming, but during stress or post-stress [12]. In the scientific literature, the priming agents are classified into three major categories: chemical agents, biostimulants and nanomaterials [11].

Phytohormones and plant growth regulators are characterized with high stress-protective potential due to their natural aptitude for subtle but prompt regulation of numerous cellular activities, thus controlling tightly multiple physiological processes [14,15,16,17,18]. Auxins, abscisic acid, ethylene, cytokinins, jasmonates, salicylic acid, brassinosteroids, strigolactones, polyamines and melatonin are able to fine tune plant reactions to abiotic stress including water shortage [14,15,19,20,21,22,23,24,25,26,27].

Melatonin (N-acetyl-5-methoxytryptamine) is a plant metabolite, derived from tryptophan, acknowledged as a master plant growth regulator with biostimulating properties [28,29,30,31,32,33,34]. The protective potential of melatonin is also attributed to its high ROS scavenging efficiency [14,33]. Pre-treatment with melatonin has been attracting research attention due to its multilevel positive effects on various plants such as alfalfa, maize, coffee, barley, wheat, etc., subjected to shortage of water [2,25,29,35,36,37,38]. Melatonin seed priming was noted as a promising method to improve drought tolerance as well as other abiotic constraints in different plants [29,39]. Studying the physiological effect of its application in hydroponic wheat cultures with or without PEG-6000, Li et al. [25] assumed that melatonin is involved in intricate mechanism of physiological adjustments, which depends on the cultivar drought tolerance. Comparing different methods of melatonin application in maize or soybean subjected to soil drought, Huang et al. [36] and Imran et al. [40] concluded that in maize and soybean, respectively, the root irrigation resulted in protection that is more effective than the leaf-spraying, considering photosynthesis and the antioxidant defense system. A recent meta-analysis of photosynthetic efficiency and abiotic stress mitigation in wheat considered the positive effects of melatonin as a promising plant growth regulator for stress management [41]. Most researchers have explored seed [2,29,35,39,42] or foliar [2,35,43,44] priming with melatonin. However, research of the biochemical response examining root application of melatonin via soil drenching recently arose [44,45,46,47,48,49,50,51], but few reports on wheat varieties were found in the scientific literature [45,50,51]. Multiple root supplementation with 500 µM melatonin was reported to act positively on the biosynthesis of reduced glutathione and ascorbate with impact on the ascorbate–glutathione cycle in wheat under moderate and severe water deficit [45]. Recently, our research group reported the physiological effects of 75 µM melatonin application before and after stress in wheat subjected to soil drought [50,51]. We showed that melatonin preserves biomass and regulates stress indicators, osmolyte levels, and proline biosynthesis gene expression, as well as photosynthetic parameters, maintaining them nearer to control values.

This study aimed to analyze the antioxidant performance of wheat cultivars in response to melatonin applied before and after stress provoked by soil drought. The analyses are focused on the enzymatic and non-enzymatic antioxidant defense systems, along with transcript profiling of genes coding for the major ROS scavenging enzymes. The present study contributes to the mechanistic understanding of the stress-alleviating potential of melatonin applied on drought-affected wheat before and after stress.

## 2. Results

### 2.1. Effect of Drought and Melatonin on Non-Enzymatic Antioxidant Defense Compounds

#### 2.1.1. Total Phenolics and Thiol Groups Containing Compounds

**Figure 1:**
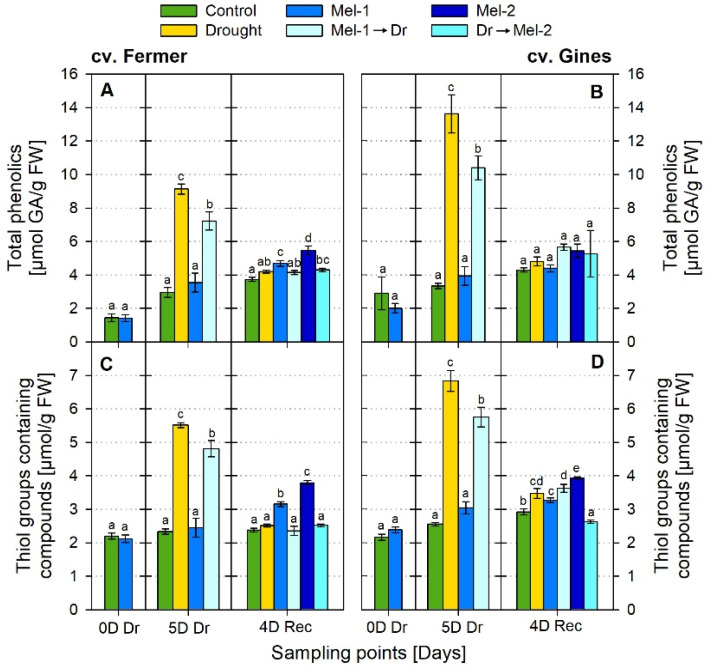
Content of total phenolics (**A**,**B**), and thiol groups containing compounds (**C**,**D**) in the wheat varieties Fermer (**A**,**C**) and Gines (**B**,**D**) before drought (0D Dr), on the fifth day of drought (5D Dr) and after four days of recovery (4D Rec). The abbreviations within the legend are as follows: Control—normally irrigated non-treated; Drought—5 days of water deficit; Mel-1—melatonin treated before drought, normally irrigated; Mel-2—melatonin treated after drought, normally irrigated; Mel-1→Dr—melatonin treated, then subjected to drought, Dr→Mel-2—subjected to drought, then melatonin treated. Error bars designate standard errors. Different letters indicate statistically significant differences at *p* ≤ 0.05 (*n* = 9).

Drought increased total phenolics (more than threefold, Figure 1A,B) as well as thiol groups containing compounds (about twofold, Figure 1C,D) compared to the controls on the fifth day of stress. Thiol groups containing compounds were still slightly enhanced on the fourth day of recovery in cv. Gines (Figure 1D) but not in cv. Fermer (Figure 1C).

Applied alone, melatonin pre-treatment (Mel-1) increased total phenolics content in Fermer (Figure 1A) only at the final sampling point. Similarly, melatonin post-treatment (Mel-2) caused a slightly higher rise than the pre-treatment, which was also detected only in cv. Fermer. Both melatonin-alone treatments (Mel-1 and Mel-2) induced thiol groups containing compounds only on the fourth day of recovery, and the highest levels in both cultivars were detected after melatonin post-treatment (Figure 1C,D).

In both wheat cultivars, melatonin pre-treatment significantly decreased the rise in total phenolics and thiol groups containing compounds (5D Dr) caused by drought (Mel-1→Dr). After recovery, the content of thiol groups containing compounds remained enhanced only in cv. Gines (Figure 1D).

On the fourth day of recovery, the melatonin post-treatment combined with drought (Dr→Mel-2) led to a minor decrease in thiol groups containing compounds, only in cv. Gines (Figure 1D), while no substantial alterations were found in total phenolics content in both cultivars.

#### 2.1.2. Glutathione Content

**Figure 2:**
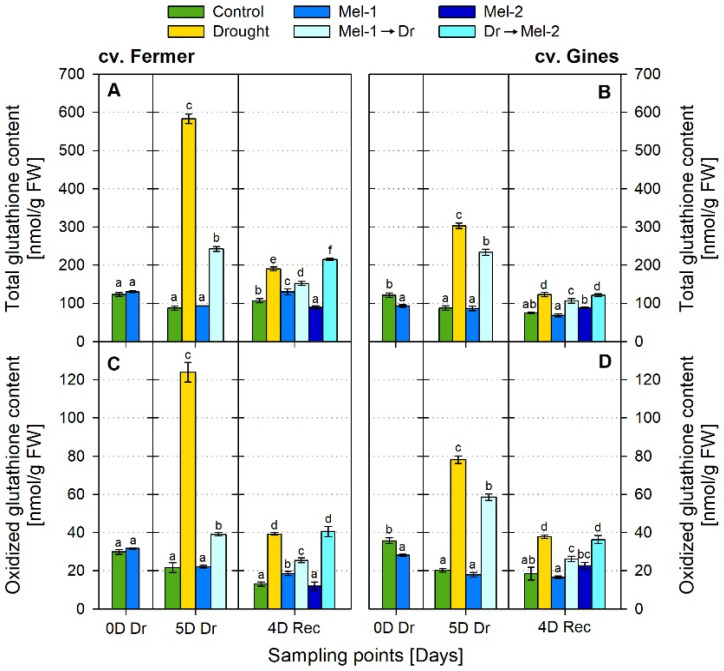
Content of total (**A**,**B**), and oxidized (**C**,**D**) glutathione content in the wheat varieties Fermer (**A**,**C**) and Gines (**B**,**D**) before drought (0D Dr), on the fifth day of drought (5D Dr) and after four days of recovery (4D Rec). The abbreviations within the legend are as follows: Control—normally irrigated non-treated; Drought—5 days of water deficit; Mel-1—melatonin treated before drought, normally irrigated; Mel-2—melatonin treated after drought, normally irrigated; Mel-1→Dr—melatonin treated, then subjected to drought, Dr→Mel-2—subjected to drought, then melatonin treated. Error bars designate standard errors. Different letters indicate statistically significant differences at *p* ≤ 0.05 (*n* = 9).

Total glutathione (Figure 2) is the pool of reduced (GSH) plus oxidized (GSSG) fraction. Drought substantially increased the total (Figure 2A,B) and oxidized (Figure 2C,D) glutathione content in cv. Fermer (fivefold) and cv. Gines (twofold). The glutathione levels declined when water supply was resumed but remained higher than the control.

Melatonin applied alone did not cause noticeable alterations in the glutathione content, although some slight fluctuations were observed.

Melatonin pre-treatment combined with drought decreased considerably total and oxidized glutathione content in cv. Fermer as compared to the drought-only variant (Figure 2A,C) but this effect was less pronounced in the tolerant cv. Gines (Figure 2B,D). The observed difference faded away with time, and glutathione content dropped to the control values at the final sampling point. Melatonin post-treatment combined with drought significantly increased total and oxidized glutathione content compared to control, and the levels were comparable with those in the drought-treated plants.

### 2.2. Effect of Drought and Melatonin on the Activity of Antioxidant Defense Enzymes and Transcript Profiling of the Corresponding Genes

#### 2.2.1. Superoxide Dismutase and Expression of Corresponding Coding Genes

**Figure 3:**
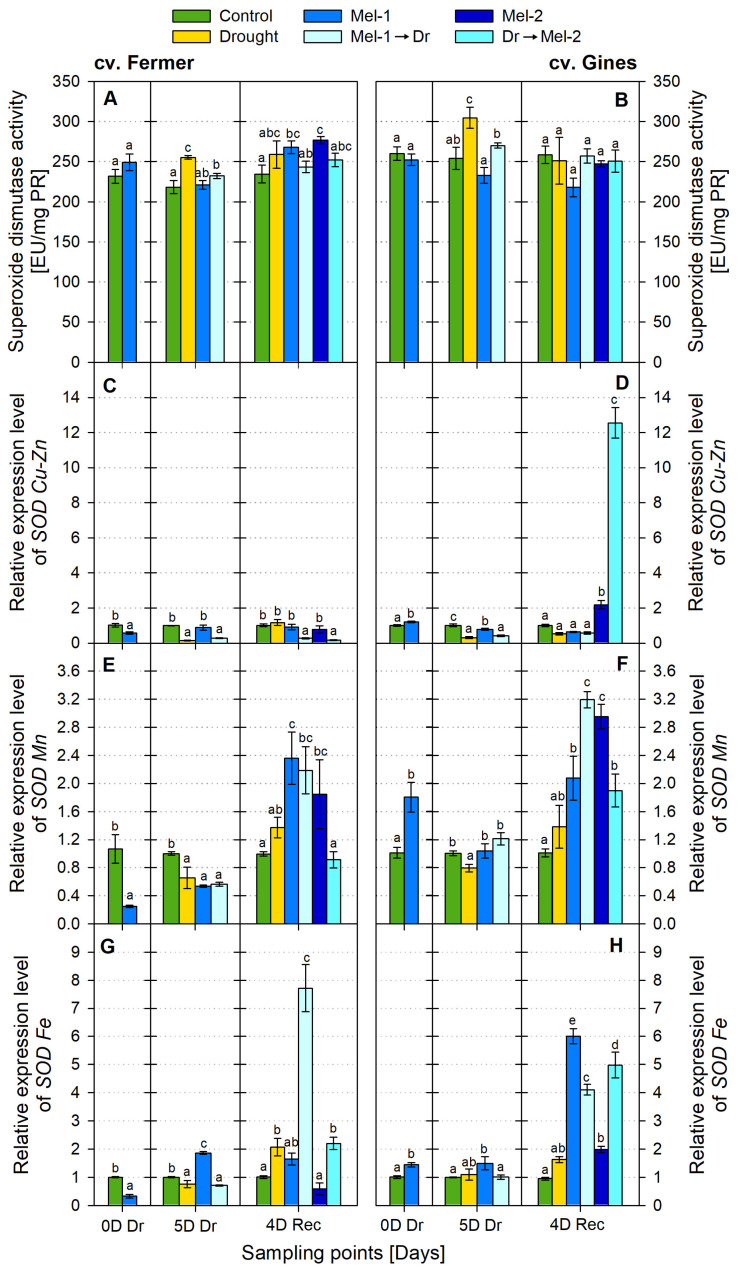
Enzymatic activity of superoxide dismutase (SOD) (**A**,**B**), and relative expression level of *SOD Cu-Zn* (**C**,**D**), *SOD Mn* (**E**,**F**) and *SOD Fe* (**G**,**H**) in the wheat varieties Fermer (**A**,**C**,**E**,**G**) and Gines (**B**,**D**,**F**,**H**) before drought (0D Dr), on the fifth day of drought (5D Dr) and after four days of recovery (4D Rec). The abbreviations within the legend are as follows: Control—normally irrigated non-treated; Drought—5 days of water deficit; Mel-1—melatonin treated before drought, normally irrigated; Mel-2—melatonin treated after drought, normally irrigated; Mel-1→Dr—melatonin treated, then subjected to drought, Dr→Mel-2—subjected to drought, then melatonin treated. Error bars designate standard errors. Different letters indicate statistically significant differences at *p* ≤ 0.05 (for panels (**A**,**B**) *n* = 9; for panels (**C**–**H**) *n* = 6).

Drought provoked a slight increase in SOD enzymatic activity (Figure 3A,B) (5D Dr), but on the fourth day of recovery the measured levels were already similar to the controls in both cultivars. *SODs* gene expression was predominantly downregulated due to water stress on the fifth day of drought. After recovery, *SOD Cu-Zn* transcripts (Figure 3C,D) reached control levels, while *SOD Mn* expression (Figure 3E,F) was above the control in both wheat varieties. A slight induction of the abundance of *SOD Fe* transcripts (Figure 3G) was recorded in cv. Fermer only during the recovery.

Melatonin-alone pre-application (Mel-1) caused slight induction of SOD enzymatic activity only in cv. Fermer on the fourth day of recovery. Moderately enhanced accumulation of *SOD Mn* transcripts (Figure 3E) was measured during the recovery in cv. Fermer due to melatonin-alone pre-treatment, despite the observed initially mild decrease in their abundance (0D Dr and 5D Dr). *SOD Fe* relative expression was considerably enhanced (sixfold) in cv. Gines (Figure 3H) due to melatonin alone pre-treatment, while *SOD Mn* expression was only slightly elevated (Figure 3F).

Melatonin pre-treatment combined with drought slightly increased SOD enzymatic activity only in cv. Fermer on the fifth day of drought, which was not reflected in the *SODs* gene expression profiles. However, on the fourth day of recovery, increased abundance of *SOD Fe* transcripts (7.5-fold) due to the combined application of melatonin pre-treatment and drought was detected in cv. Fermer. Similarly, upregulation of *SOD-Fe* (fourfold) and *SOD Mn* (more than threefold) transcripts was recorded in cv. Gines. The expression of *SOD Cu-Zn* was low but relatively stable during the whole studied period in both wheat varieties subjected to melatonin pre-treatment combined with drought.

Melatonin post-treatment applied alone raised the enzymatic activity of SOD only in cv. Fermer on the fourth day of recovery. However, the transcript abundance of *SOD Mn* was only slightly enhanced. Melatonin post-treatment applied alone led to modest upregulation of *SOD Cu-Zn* and *SOD Fe* transcript and considerable increase in *SOD Mn* expression (threefold) in cv. Gines (4D Rec).

At the final sampling point, melatonin treatment applied after drought had the opposite effect on the transcript abundance of *SOD Cu-Zn* in both wheat cultivars. The relative expression level of *SOD Cu-Zn* was downregulated in cv. Fermer, while a very strong upregulation (12-fold) was recorded in cv. Gines (Figure 3C,D). A mild induction of *SOD Fe* transcript levels due to melatonin application after drought was measured in cv. Fermer, while relatively high expression (fivefold) was recorded in cv. Gines. The post-drought treatment group did not cause alteration of SOD enzymatic activity compared to the respective control.

#### 2.2.2. Catalase and Expression of Corresponding Coding Genes

**Figure 4:**
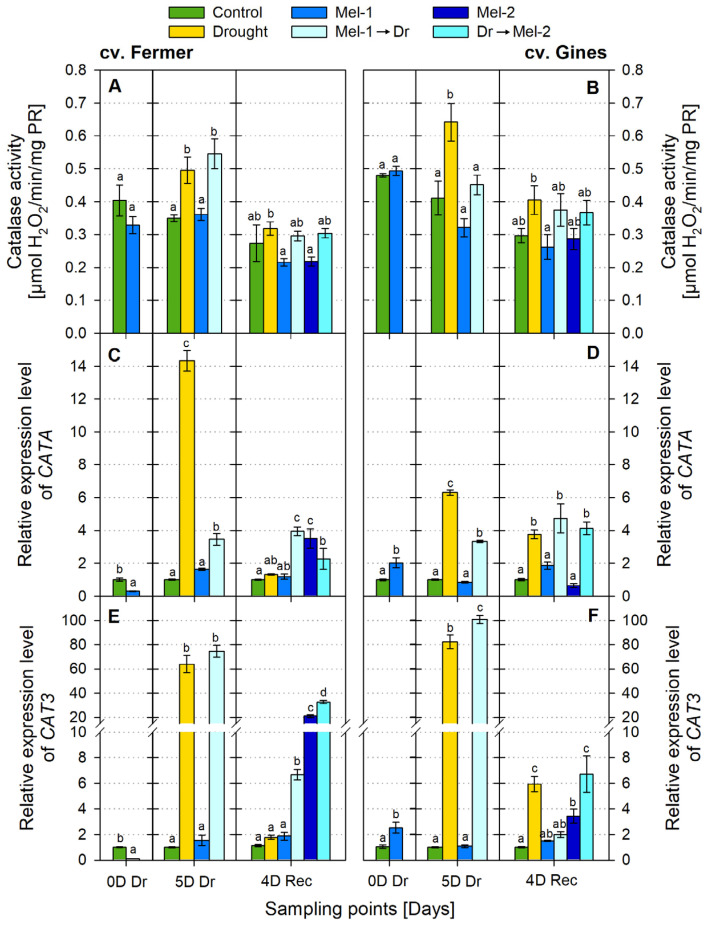
Enzymatic activity of catalase (CAT) (**A**,**B**) and relative expression level of *CATA* (**C**,**D**) and *CAT3* (**E**,**F**) in the wheat varieties Fermer (**A**,**C**,**E**) and Gines (**B**,**D**,**F**) before drought (0D Dr), on the fifth day of drought (5D Dr) and after four days of recovery (4D Rec). The abbreviations within the legend are as follows: Control—normally irrigated non-treated; Drought—5 days of water deficit; Mel-1—melatonin treated before drought, normally irrigated; Mel-2—melatonin treated after drought, normally irrigated; Mel-1→Dr—melatonin treated, then subjected to drought, Dr→Mel-2—subjected to drought, then melatonin treated. Error bars designate standard errors. Different letters indicate statistically significant differences at *p* ≤ 0.05 (for panels (**A**,**B**) *n* = 9; for panels (**C**–**F**) *n* = 6).

An induction of catalase enzymatic activity was recorded after five days of drought, particularly pronounced in cv. Gines (Figure 4A,B). The expression levels of catalase coding genes (*CATA*, *CAT3*) were considerably upregulated (14-fold and more than 60-fold, respectively) in cv. Fermer (5D Dr—Figure 4C,E). The transcript abundance of catalase coding genes in cv. Gines (Figure 4D,F) was enhanced moderately for *CATA* (6-fold) and substantially for *CAT3* (80-fold) on the fifth day of stress.

Melatonin pre-treatment alone did not alter CAT activity (Figure 4A,B). However, on the first measurement point (0D Dr), it led to a slight decrease in *CATA* and *CAT3* transcript levels in cv. Fermer (Figure 4C,E), while provoking a noticeable augmentation of catalase transcripts in cv. Gines (Figure 4D,F).

Melatonin pre-treatment combined with drought increased catalase enzymatic activity on the fifth day of drought only in cv. Fermer. Correspondingly, upregulation of catalase-coding genes was also observed. *CAT3* transcript levels decreased with time in cv. Fermer (Figure 4E). However, stable but moderate upregulation of *CATA* transcript levels were registered throughout the experimental period (Figure 4C). In cv. Gines (Figure 4D,F) the expression levels of catalase genes were altered in a similar manner to cv. Fermer (Figure 4C,E).

Melatonin post-treatment alone or combined with drought considerably increased the transcript abundance of *CAT3* in cv. Fermer (more than 30-fold), whereas the induction of *CATA* and *CAT3* expression in cv. Gines was moderate.

#### 2.2.3. Guaiacol Peroxidase and Expression of Corresponding Coding Gene

**Figure 5:**
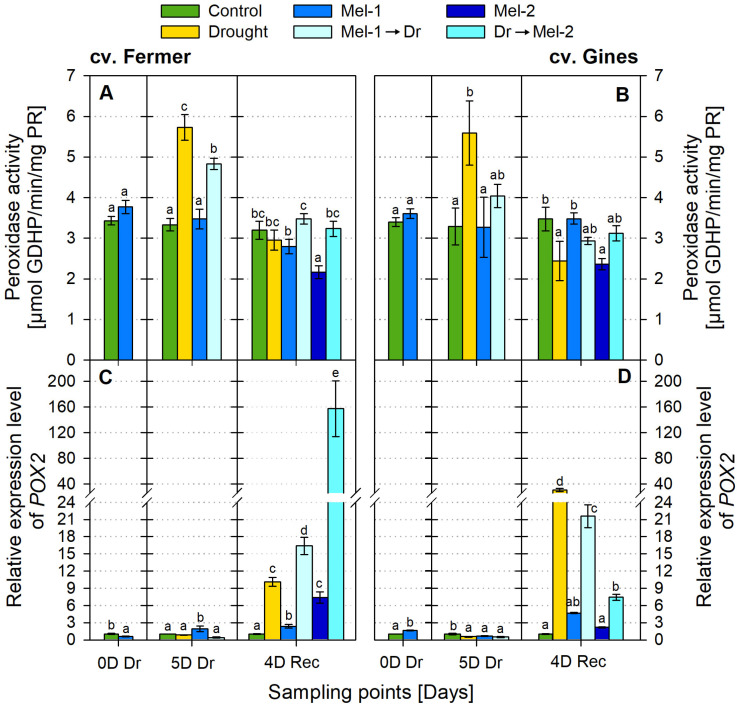
Enzymatic activity of peroxidase (POX) (**A**,**B**), and relative expression level of *POX2* (**C**,**D**) in the wheat varieties Fermer (**A**,**C**) and Gines (**B**,**D**) before drought (0D Dr), on the fifth day of drought (5D Dr) and after four days of recovery (4D Rec). The abbreviations within the legend are as follows: Control—normally irrigated non-treated; Drought—5 days of water deficit; Mel-1—melatonin treated before drought, normally irrigated; Mel-2—melatonin treated after drought, normally irrigated; Mel-1→Dr—melatonin treated, then subjected to drought, Dr→Mel-2—subjected to drought, then melatonin treated. Error bars designate standard errors. Different letters indicate statistically significant differences at *p* ≤ 0.05 (for panels (**A**,**B**) *n* = 9; for panels (**C**,**D**) *n* = 6).

The enzymatic activity of peroxidase was markedly increased due to drought on the fifth day of stress in both studied varieties, but during the recovery it reached the control value in cv. Fermer (Figure 5A) and dropped below the control in cv. Gines (Figure 5B). The expression levels of guaiacol peroxidase (*POX2*) were upregulated after recovery, by 10-fold in cv. Fermer (Figure 5C) and about 40-fold in cv. Gines (Figure 5D).

No substantial alterations in peroxidase activity were observed after melatonin pre-treatment (Figure 5A,B). A slight decrease in the levels of *POX2* transcript abundance was detected in cv. Fermer at the first measurement point (Figure 5C) (0D Dr). Then, an increase was registered on the fifth day of drought. An opposite tendency was observed in cv. Gines (Figure 5D).

If compared to drought alone, melatonin pre-treatment reduced the drought-increased peroxidase activity in both cultivars (Figure 5A,B) but the effect was stronger in cv. Gines (5D Dr). Interestingly, a comparable upregulation of *POX2* in both wheat varieties (Figure 5C,D) was observed only after four days of recovery.

Peroxidase enzymatic activity decreased due to melatonin-alone post-treatment, but when applied in combination with drought, the activity was comparable to the respective controls (Figure 5A,B). Melatonin-alone post-treatment led to increased transcript abundance of *POX2* (7-fold) only in cv. Fermer (Figure 5C). Melatonin treatment after drought substantially upregulated *POX2* expression in cv. Fermer (near 160-fold), but only a moderate effect was observed in the same treatment group of cv. Gines plants.

#### 2.2.4. Glutathione Reductase and Expression of Corresponding Coding Gene

**Figure 6:**
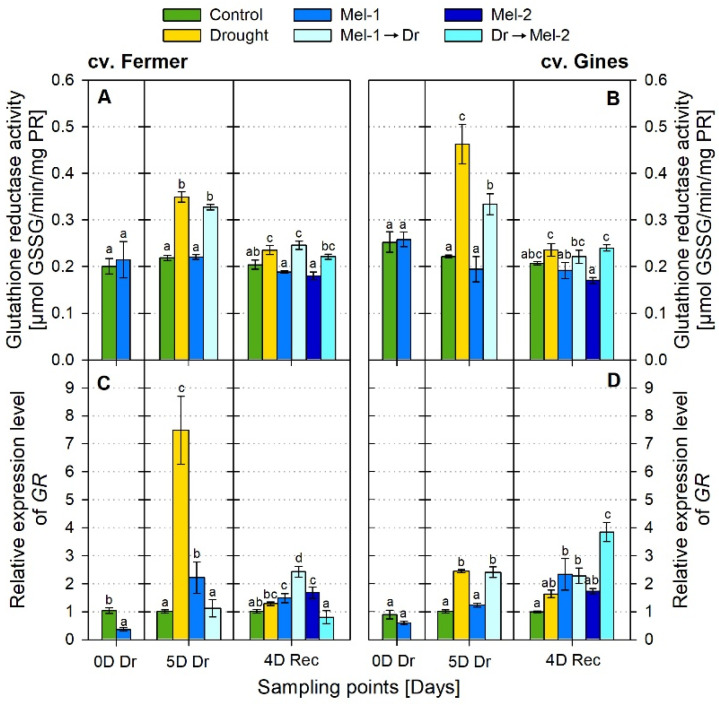
Enzymatic activity of glutathione reductase (GR) (**A**,**B**), and relative expression level of *GR* (**C**,**D**) in the wheat varieties Fermer (**A**,**C**) and Gines (**B**,**D**) before drought (0D Dr), on the fifth day of drought (5D Dr) and after four days of recovery (4D Rec). The abbreviations within the legend are as follows: Control—normally irrigated non-treated; Drought—5 days of water deficit; Mel-1—melatonin treated before drought, normally irrigated; Mel-2—melatonin treated after drought, normally irrigated; Mel-1→Dr—melatonin treated, then subjected to drought, Dr→Mel-2—subjected to drought, then melatonin treated. Error bars designate standard errors. Different letters indicate statistically significant differences at *p* ≤ 0.05 (for panels (**A**,**B**) *n* = 9; for panels (**C**,**D**) *n* = 6).

The main enzymatic function of GR is to convert oxidized glutathione (GSSG) into reduced glutathione (GSH), which is an NADPH-dependent process. Drought stress induced the enzymatic activity of glutathione reductase (GR) in cv. Fermer (Figure 6A) and more significantly (twofold) in cv. Gines (Figure 6B). Drought also increased the relative transcription levels of *GR*. In cv. Fermer (Figure 6C), it was 7.5-fold higher than the control, while only a moderate increase in the expression was observed in cv. Gines (Figure 6D). Melatonin-alone treatment (pre- or post-stress) did not cause considerable alterations in the levels of enzymatic GR activity and *GR* transcripts abundance.

Melatonin pre-treatment combined with drought did not alter GR activity in cv. Fermer, while in cv. Gines it was significantly reduced (Figure 6A,B). *GR* transcript levels on the fifth day of drought remained similar to the control in the melatonin pre-treated cv. Fermer plants, while in the same cv. Gines group they were comparable with the levels measured in the drought-stressed-only individuals (Figure 6C,D).

During the recovery, melatonin pre-treatment combined with drought kept *GR* transcripts slightly upregulated in both cultivars.

Melatonin post-treatment applied alone or in combination with drought did not alter enzymatic activity of GR in both varieties (Figure 6A,B). A slight increase in *GR* expression was observed only in cv. Fermer due to melatonin-alone post-treatment (Figure 6C). However, a moderate increase (fourfold) was observed in cv. Gines after combined application of melatonin post-treatment with drought (Figure 6D).

## 3. Discussion

There are three basic experimental setups to model drought stress, such as soil-based, hydroponic-based or agar-based culture (containing non-ionic carbohydrate osmolytes as sorbitol and mannitol, or PEG) [52]. Many investigations with root melatonin application have studied drought stress using a PEG experimental setup [25,49,53,54]. The soil-based experimental model of drought stress most accurately reflects the naturally occurring water deficiency. Some authors have shown that the beneficial effects of melatonin soil drenching on the antioxidative system in wheat [45] and maize [47] are more pronounced under moderate drought than under severe stress. Our study is consequent to previous research [50,51] employing a model, which closely mimics water soil deficiency to evaluate the effects of melatonin pre-treatment and post-treatment via roots on the modulation of moderate soil drought stress response of the drought-tolerant cultivar Gines and the less drought-tolerant cultivar Fermer [55,56]. We found that the levels of stress markers (MDA, hydrogen peroxide, electrolyte leakage), osmolyte content, proline biosynthesis transcript levels [50], photosynthesis-related parameters, and phenotypic characteristics [51] were all kept closer to the control levels during the stress period when soil melatonin is supplemented prior to the development of water deficit. The physiological consequences of both melatonin treatments (Mel-1→Dr/Dr→Mel-2) facilitated the recovery of wheat plants when the water supply was resumed. The post-drought application Dr→Mel-2 exhibited a similar to, but weaker effect than, pre-drought treatment Mel-1→Dr [50,51]. The current research extends our previous investigations, with focus on the activities of the main enzymes of the antioxidant defense (superoxide dismutase, catalase, peroxidase, glutathione reductase, and the expression of their related genes), and the non-enzymatic antioxidant defense such as total phenolics, thiol group containing compounds, and glutathione in wheat leaves.

In general, the action of ROS and antioxidants (non-enzymatic and enzymatic) has a significant role not only in the redox status of cells but also in mechanisms related to epigenetic changes, known to be implicated in ontogenesis, plant response to stress and many other processes [10,57]. In actuality, the positive effect of hormonal priming, including the generation of stress memory, is often associated with epigenetic modifications and the observed adaptation to stress [10,11].

Current results extend the knowledge on melatonin potential as a drought-stress-alleviating compound. The positive activation of antioxidant defense through melatonin root application, both prior to and following soil drought, provides new insights into environmentally safe strategies for mitigating the effects of water deficit in wheat.

Superoxide dismutase as a part of the enzymatic antioxidants is acknowledged as the first defense line toward ROS, which catalyzes dismutation of superoxide to hydrogen peroxide and oxygen in different cell compartments [5,6,58]. The classification of the enzymatic family of SOD is based on their metal cofactors (Cu-Zn, Fe, Mn), and the sub-cellular localization of isoenzymes gene expression may vary in plants [58,59]. The SOD Mn was noted to be predominantly located in the mitochondrion and peroxisomes, the SOD Fe in the chloroplasts, and SOD Cu-Zn is mainly active in the chloroplasts and the cytosol. The moderate level of drought stress applied might be the reason for the observed lack of intensive changes in enzymatic activity of SOD and has been reported before in maize [47] and wheat [60]. A possible explanation could be also a different engagement of the SOD isoenzymes and the corresponding isogenes. In wheat, the expressions of *SOD Cu-Zn* and *SOD Mn* were reported to increase under water shortage [59]. However, other authors [61] showed that the mitochondrial *SOD Mn* transcripts were drought-inducible, but *SOD Cu-Zn* were expressed at the recovery stage. Interestingly, the slight stress-induced SOD activity was not accompanied by activated expression of any of the monitored SOD-coding genes. Activation of *SOD* transcripts in combination with melatonin treatment groups was observed mainly during the recovery period. These results suggest that melatonin might enhance *SOD* expression, which would be beneficial for ROS scavenging. The obtained data imply the particular importance of both *SOD Cu-Zn* and *SOD Mn* transcript abundance for improved adaptation capacity in the drought-tolerant cv. Gines. As there is a time-shift in application of melatonin before and after the stress, the possibility of sequential upregulation of SOD-coding genes (initially *SOD Mn* followed by *SOD Cu-Zn*) is not excluded when the combination of drought and melatonin was applied to the more tolerant wheat variety. Additionally, the slight and consistent (basal) induction of *SOD Fe* transcripts observed under both combined melatonin treatments (pre- and post-drought) is relevant because of their chloroplast location, as it has been already acknowledged that photosynthesis is as a major source of ROS [5]. Moreover, the downregulation of the SOD transcripts was found to be more consistent in the less drought-tolerant cv. Fermer than in cv. Gines. The results are in line with previous data that showed higher disruption of photosynthetic parameters for cv. Fermer than cv. Gines [51]. Indeed, Baranova et al. [62] demonstrated that one of the strategies for cold acclimation counts on the protection of the photosynthetic structures via insertion of the heterologous *SOD Fe* gene into the tobacco genome. In general, the induction of all types of SOD-coding genes in cv. Gines, when subjected to combined melatonin and drought treatment, probably ensures higher acclimation prospects. The upregulation of SOD-coding genes by melatonin pre-treatment are consistent with our previous reports on photosynthesis performance and hydrogen peroxide content [50,51]. However, cv. Fermer probably uses another SOD-related adaptation strategy when water shortage is combined with melatonin pre-treatment: a substantial induction in *SOD Fe* transcripts, located in chloroplasts, and slight activation of *SOD Mn* (located in mitochondria and peroxisomes) expression at recovery phase. Thus, the results obtained might be associated with differences in adaptation potential and strategies of the investigated wheat varieties when melatonin is applied in combination with drought.

Catalase and peroxidases are important enzymes for H_2_O_2_ detoxification. The first one is known as the primary respondent to oxidative stress, and the second one is essential in keeping the delicate balance of hydrogen peroxide levels within cells [5,63]. The enzymatic activities of CAT and POX obtained on the fifth day of stress are in concert with the H_2_O_2_ levels estimated in our previous study [50]. Similarly, the relative expression of catalase coding genes (*CATA* and *CAT3*) is in line with the detected peroxide content [50]. The catalase activity was markedly induced by drought in cv. Gines. A distinct upregulation of *CAT3* persisted during stress and after recovery in the tolerant cultivar. In contrast, the measured slight increase in enzymatic activity and severe but time-limited drought-inducible upregulation of the studied catalase genes (*CATA* and *CAT3*) in the less drought-tolerant variety Fermer appear inconsistent. However, it should be noted that there are ten *CAT* genes in the wheat genome, and the analysis of their upstream promoter regions confirms a complex multilevel regulation of their expression [64,65]. We assume that other catalase-coding genes, in addition to the studied *CATA* and *CAT3*, could be also engaged forming a complex interplay with the master plant growth regulator (viz., melatonin) shaping up the drought response. Our results are in line with the documented induction of *CAT* upregulation as a rapid response of intervarietal substitution wheat lines to short-term PEG stress [66]. The combined application of melatonin with drought showed the distinct involvement of *CAT3* transcripts in wheat response, particularly in cv. Fermer, together with a clear time-shift noticed due to the different time point of melatonin treatment before and after stress. Further, the induction of CAT enzymatic activity, observed in cv. Fermer following melatonin pre-treatment under drought, reaching levels comparable to those observed in drought-only treatment (5D Dr) could indicate a cultivar specificity in the melatonin-mediated adaptation. The result obtained supports the reported increase in CAT activity by melatonin root application in maize [47] and barley [44].

Guaiacol peroxidase enzymatic (POX) activity was distinctly drought-inducible in both studied wheat cultivars. The result corresponded well with the observed high content of phenolic compounds as the polyphenols are the usual reductant of POX used to scavenge H_2_O_2_. The decrease in POX enzymatic activity during the recovery period in cv. Gines might be attributed to the fine-tuning of the enzyme in H_2_O_2_ regulation, which is consistent with relatively higher drought tolerance of the variety. The marked expression of *POX2* transcript abundance identified only during recovery does not correspond to the measured enzymatic activity of the relevant enzyme. The involvement of both intracellular and apoplastic forms of guaiacol peroxidase [67] and the existing multigene family of peroxidase genes in *T. aestivum* [68,69] could explain the observed discrepancy. Additionally, the upregulation of *POX2* transcripts observed in all drought-treated variants of both cultivars during the recovery period suggests that the plants may have entered a state that could improve their readiness to cope with future stress events. This observation could explain the previously reported decrease in stress markers malondialdehyde and electrolyte leakage [50], and the better phenotype performance of melatonin-treated (Mel-1→Dr/Dr→Mel-2) versus drought-treated wheat cultivars Fermer and Gines [51]. Furthermore, a similar increase in transcript levels of peroxidase was reported in pea during recovery following drought [70]. The distinct differences between varieties, due to melatonin pre- and post- application in combination with drought, could be explained by different drought tolerance of the cultivars. The induction pattern of *POX2* transcripts in the less drought-tolerant cv. Fermer after combined treatment suggests an enhanced potential of the antioxidant defense due to melatonin application in this particular variety. The expression pattern of *POX2* in cv. Gines is consistent with trends reported by other authors in different drought-tolerant wheat cultivars [66,71]. It could be speculated that cv. Gines has a better intrinsic potential for adaptation to limited water supply; therefore, the melatonin treatment did not additionally enhance this particular component of antioxidant defense.

Glutathione reductase (GR) and ascorbate peroxidase, together with the ascorbate and glutathione, are major contributors to ascorbate–glutathione-related defense in plants [5,6]. Appropriate levels of the ascorbate–glutathione pathway components are important for plant growth, development and survival under stress because of their buffering potential and protective role against reactive oxygen species. Two molecules of reduced glutathione (GSH) form glutathione disulfide (GSSG) or an oxidized glutathione. Stress modifies the balance of ascorbate–glutathione pathway participants in cellular compartments and disorders the regular redox state within organelles [72,73]. However, the electron transfer is a reversible process: GR catalyzes the reduction in GSSG to the reduced glutathione via NADPH dependent reaction, and GSH participates in the ascorbate–glutathione cell cycle. In general, an amplified amount of GSH has a positive function in plant tolerance and assists to diminish oxidative stress, but an increase in GSSG indicates a disturbance in the redox balance [74,75]. Thus, the observed boost of oxidized glutathione content due to drought (5D Dr) is an expected negative consequence, and its diminution after resuming watering indicates that plants attempt to maintain the redox homeostasis. However, melatonin pre-treatment clearly reduced the stress-induced accumulation of oxidized glutathione, thereby facilitating the plants to shift the redox state closer to the control in both cultivars. The high activity of the GR enzyme supplements the above statement. Additionally, the consistently enhanced enzymatic activity of GR due to combined melatonin pre-treatment, particularly in cv. Fermer, highlights the importance of the ascorbate–glutathione cycle in the induced enhancement of plant tolerance potential. Despite the equal enzymatic activity of GR in wheat subjected to drought and to combined pre-treatment, cv. Fermer demonstrated substantially lower GSSG content than the stressed-only plants, indicating that melatonin application could be beneficial for strengthening the antioxidant defense, particularly in the less tolerant cultivar. In contrast, the reduced GR activity in cv. Gines at 5D Dr after the combined treatment, relative to drought-only plants, was accompanied by relatively high GSSG content, which suggests that glutathione may not be the principal component involved in melatonin-mediated alleviation of drought stress in this cultivar. The effect of melatonin addition after stress on the oxidized glutathione content and on the activity of GR is similar to the plants subjected to drought only. This could indicate that pre-treatment is more effective than post-treatment in keeping glutathione homeostasis. The enhanced thiol groups containing compounds observed at recovery in cv. Gines only probably suggest the involvement of other sulfur-based reactive molecules, different from glutathione in melatonin-induced alleviation of drought effects.

In general, the observed difference between enzyme activity and transcript abundance likely reflects the fact that drought and recovery activate antioxidant defenses at different regulatory levels and with different kinetics. We found that gene expression of SOD, CAT, and POX often changed more strongly than the corresponding enzyme activities. Similar uncoupling between antioxidant enzyme activity and gene expression has been reported in wheat under drought [76,77], where both crop genotype and stress severity influenced the magnitude of the response, and drought/rewatering further altered antioxidant dynamics during recovery [60]. Drought and recovery are different physiological states, and transient transcriptional peaks are not always matched immediately by the enzyme activity, as we observed. The pattern of induction or suppression of enzyme activity and gene expression could be additionally complicated by the action of melatonin, which might shift the activity and transcript profile of antioxidant defense [78]. All of this implies that the modulatory effect of melatonin on wheat antioxidant defense demonstrated in the current study is a multilayer process.

## 4. Materials and Methods

### 4.1. Plant Material and Experimental Model

Two Bulgarian hexaploid winter wheat (*Triticum aestivum* L.) varieties selected in the Institute of Plant Genetic Resources “K. Malkov” (Sadovo, Bulgaria) were used. The cultivars were phylogenetically analyzed previously [79], and cv. Gines was previously reported to be more drought-tolerant than cv. Fermer [55,56]. A total of 20 seeds were sown in 500 cm^3^ pots filled with 560 g substrate of leached meadow cinnamon soil:sand (3:1) and grown under controlled conditions (14/10 h photoperiod, 200 μmol/m^2^/s photon flux density, 21 °C/19 °C day/night temperatures, 60% relative air humidity) in a growth chamber. Plant treatments were divided into 6 groups, each consisting of 6 pots (120 plants per treatment). Then, 17-day-old seedlings at BBCH stage 12 (fully developed second leaf) were exposed to drought stress by withholding watering for 5 days. The control plants were irrigated to sustain 75% of the field moisture capacity. The water stress was terminated by restoration of the normal irrigation regime, and plants had a four-day recovery period. Part of the control plants and those subjected to drought obtained 10 mL of 75 µM aqueous melatonin solution added to the irrigation water for each pot, applied either 24 h before or after the stress. The melatonin treatment protocol was chosen based on the positive outcome obtained in previous research [80,81].

### 4.2. Biochemical Analyses

The above-ground part of three plants was pooled and collected for analyses before drought (0D Dr), on the fifth day of water shortage (5D Dr) and four days after drought during the recovery period (4D Rec). The collected samples were frozen in liquid nitrogen and preserved at −80 °C until analyses. The extraction procedures were performed at 4 °C. All chemicals used in the biochemical analyses were purchased from Merck (Burlington, MA, USA). The measurements were conducted on a UV/VIS Multiskan Spectrum spectrophotometer with a microplate reader (Thermo Electron Corporation, Vantaa, Finland) and on a Shimadzu UV/VIS i1900 spectrophotometer (Shimadzu, Kyoto, Japan). A refrigerated Sigma 2-16K centrifuge (SciQuip, Wem, UK) was used to obtain the relevant supernatants.

#### 4.2.1. Enzymatic Activity Quantification

Approximately 200 mg of plant material was homogenized in 3 mL ice-cold 100 mM potassium phosphate buffer (pH 7.0) containing 1 mM ethylenediaminetetraacetic acid and 1% polyvinylpyrrolidone. The homogenate obtained was centrifuged at 15,000× *g* and the supernatant was used for the determination of different enzymatic activities. The activity of superoxide dismutase enzyme was assessed following the inhibition of the photochemical reduction of nitroblue tetrazolium at 560 nm. One unit of SOD corresponds to the amount of enzyme required to cause 50% inhibition of the reaction [82]. The method of Aebi [83] was used to determine catalase activity: the degradation rate of H_2_O_2_ was followed at 240 nm. Guaiacol was used as an electron donor to assess peroxidase activity [84], and the change in absorbance at 470 nm was monitored. To estimate the activity of glutathione reductase, the rate of reduction of oxidized glutathione was measured at 412 nm [85]. The protein content was determined by a standard curve prepared with bovine serum albumin [86].

#### 4.2.2. Endogenous Glutathione Quantification

The endogenous glutathione concentrations were estimated according to the enzymatic recycling procedure described by Gronwald et al. [87]. Standard curves were used to calculate the amounts of total (sum of reduced (GSH), and oxidized (GSSG)) glutathione and GSSG form by monitoring the change in optical density at 412 nm.

#### 4.2.3. Total Phenolics and Thiol Groups Content

Approximately 250 mg of leaf material was grinded in 0.1% cold trichloroacetic acid and was centrifuged for 30 min (15,000× *g*, 4 °C). The resulted supernatant was used for analyses of the non-enzymatic antioxidants.

The content of free thiol containing compounds was determined with Ellman’s reagent, and the quantity was calculated by using the molar extinction coefficient of 13.6 mM^−1^cm^−1^ [88].

Total phenols content was determined with Folin–Ciocalteau reagent supplemented with sodium carbonate, and the absorbance was read at 725 nm according to the method of Swain and Goldstein [89]. The total phenolic content was calculated using a standard curve prepared with known amounts of gallic acid.

### 4.3. RT-PCR Analysis

The GeneJET Plant RNA Purification Kit (Thermo Scientific, Waltham, MA, USA) was used for total RNA extraction. The RNA concentration of samples was measured on a micro-UV–VIS spectrophotometer, Nano Drop 2000 (Thermo Scientific, Basel, Switzerland). Total RNA of 1 μg was used to synthesize copy DNA using the Scriptase RT—cDNA Synthesis Kit (GENAXXON Bioscience, Ulm, Germany) according to the instructions of the manufacturer. Evaluation of the gene transcript abundance was done by quantitative real-time RT-PCR (qRT-PCR) in a ‘PikoReal’ Real-Time PCR System (Thermo Scientific, Basel, Switzerland) using a 2X GreenMasterMix No ROXTM (GENAXXON Bioscience, Ulm, Germany). The qPCR analysis was performed using the following described program settings: 95 °C for 15 min and 45 cycles of 95 °C for 15 s followed by 55–60 °C for 30 s. The melting curve analysis of the PCR amplicons was conducted with a temperature range of 60–95 °C in 0.2 °C increments for 60 s.

The transcripts coding for antioxidant enzymes were the following: chloroplast *SOD Cu-Zn* (*U69632.1*), chloroplast *SOD Fe* (*LOC101290631*), mitochondrial *SOD Mn* (*LOC542833*), catalase isozyme 2 *CAT3* (*LOC100682478*), catalase *CATA* (*LOC543316*), glutathione reductase *GR* (*GR305072*), and class III peroxidase *POX2* (*LOC543313*); all are available in the database of the National Center of Biotechnology Information (NCBI). The expression of the target genes was normalized using α-tubulin (*α-TUB*, *U76558.1*), 18S ribosomal RNA (*18S RNA*, *LOC123171822*), and elongation factor-1 α (*EF-1 α*, *LOC123123039*) as reference genes, and the relative expression was evaluated using the ΔΔCq method [90]. The oligonucleotides used for the RT-qPCR analyses are presented in Table 1.

### 4.4. Statistical Analysis

The data presented are derived from three independent biological experiments. Samples were collected in triplicate for the biochemical and in duplicate for molecular assays. Each sample consisted of pooled material from three plants. The data represent average values with standard error (SE). To assess the significant differences between treatments in each experimental group, one-way ANOVA with Duncan’s post hoc multiple range test was used (*p* ≤ 0.05).

## 5. Conclusions

The antioxidant defense (both enzyme and non-enzyme components) is activated by drought, showing some variety-specific patterns. In the drought-tolerant variety Gines, significant increase in total phenolics and thiol containing compounds, CAT and GR enzyme activity along with *CATA*, *CAT3* and *POX2* transcripts was observed. On the other hand, in the less drought-tolerant variety Fermer, the most considerable increase in the parameters studied was found in the transcript levels of *CAT3*, *CATA* and *GR*, and in the glutathione pool. Fewer components of the antioxidant defense machinery were activated in the more sensitive cv. Fermer under drought stress.

Melatonin pre-treatment decreased almost all of the stress-induced parameters studied, suggesting a sustained antioxidant adjustment. Interestingly, in cv. Fermer the parameters most substantially affected by drought were also the ones that were significantly alleviated by the application of melatonin, except *CAT3*, while in the drought-tolerant variety Gines, melatonin pre-treatment significantly modulated only the activity of CAT and GR, and *CATA* transcript expression. It could be speculated that melatonin pre-treatment differentially influenced the components of the antioxidant defense in both cultivars depending on their potential to cope with stress.

Finally, comparing the pre- and post-drought melatonin application, it could be summarized that both treatment approaches produced similar effects on non-enzymatic antioxidants and antioxidant enzyme activity in both cultivars during the recovery period. However, the post-treatment specifically increased transcript expression depending on drought tolerance of the cultivars: SOD transcripts in cv. Gines, and catalase and peroxidase transcripts in cv. Fermer. This implies that melatonin application could adjust the diverse transcriptional response of the antioxidant system so that it remains activated even after rewatering.

**Figure 7:**
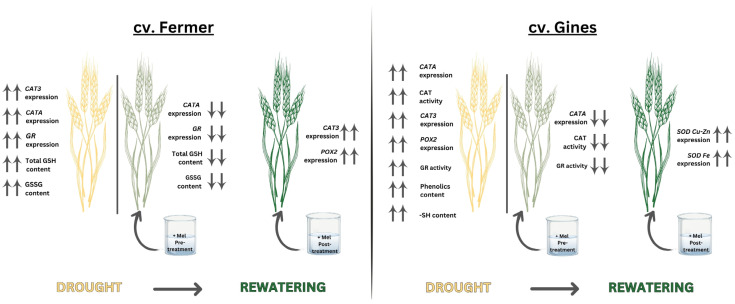
Schematic presentation of the antioxidant defense parameters most influenced by drought and melatonin treatment in two winter wheat cultivars differing in their drought tolerance.

Overall, the examined Bulgarian wheat cultivars exhibited differential physiological plasticity in the modulation of antioxidant defense systems in response to melatonin treatment, with better efficiency observed when the treatment preceded drought stress (Figure 7).

Along with its role in modulating plant stress responses, melatonin also acts as an integrator of multiple hormonal signals in plants [91]. In this context, further investigation of the endogenous levels of other major plant growth regulators, in the same experimental model, would contribute to a deeper understanding of the modulatory effects of pre- and post-application of melatonin in wheat subjected to drought.

## Figures and Tables

**Table 1:** Primer pairs used in the qRT-PCR analyses.

Gene Name	Locus	Forward Primer (5′-3′)	Reverse Primer (5′-3′)
*SOD Cu-Zn*	*U69632.1*	ttaacccaaacggcctgacacat	caacaaacgctctcccaacaactg
*SOD Fe*	*LOC101290631*	gtctggttgggtttggcttgtctt	ttcgcctgtcatccttgtaatcca
*SOD Mn*	*LOC542833*	cgccacctacgtcgcccactac	acatgaccgccgccgttgaa
*CAT3*	*LOC100682478*	caccctcgtcggcggcaagaac	cacgggctggagggggacgag
*CATA*	*LOC543316*	gggagccagtgcaaagggattc	cacggtcatgcacaacggtagaga
*POX2*	*LOC543313*	gcggtgacaccaacatcaac	gtccaggttctccaggttgg
*GR*	*GR305072*	gttgaagtcacccagccaga	tccgccaccaagaatcacag
*α-TUB*	*U76558.1*	ttctcccgcatcgaccacaagttt	tcatcgccctcatcaccgtcc
*18S RNA*	*LOC123171822*	tacctggttgatcctgccagt	caatgatccttccgcaggttcac
*EF-1 α*	*LOC123123039*	cagatcggcaacggctac	gagaaggtctccaccaccat

## Data Availability

Research data are available upon request from the corresponding author.
